# Copper signalling: causes and consequences

**DOI:** 10.1186/s12964-018-0277-3

**Published:** 2018-10-22

**Authors:** Julianna Kardos, László Héja, Ágnes Simon, István Jablonkai, Richard Kovács, Katalin Jemnitz

**Affiliations:** 1 Functional Pharmacology Research Group, Institute of Organic Chemistry, Research Centre for Natural Sciences, Hungarian Academy of Sciences, Magyar Tudósok körútja 2, Budapest, 1117 Hungary; 2 Institute of Neurophysiology, Charité-Universitätsmedizin, Berlin, Germany

**Keywords:** Redox disproportionation and speciation of copper, Dynamic copper pool, Copper-rich aggregates, GSH/GSSG ratio, Copper chelate therapy, Neuro-glia coupling

## Abstract

Copper-containing enzymes perform fundamental functions by activating dioxygen (O_2_) and therefore allowing chemical energy-transfer for aerobic metabolism. The copper-dependence of O_2_ transport, metabolism and production of signalling molecules are supported by molecular systems that regulate and preserve tightly-bound static and weakly-bound dynamic cellular copper pools. Disruption of the reducing intracellular environment, characterized by glutathione shortage and ambient Cu(II) abundance drives oxidative stress and interferes with the bidirectional, copper-dependent communication between neurons and astrocytes, eventually leading to various brain disease forms. A deeper understanding of of the regulatory effects of copper on neuro-glia coupling via polyamine metabolism may reveal novel copper signalling functions and new directions for therapeutic intervention in brain disorders associated with aberrant copper metabolism.

## Background

Copper is a generally utilized heavy metal [1] with a toxic limit beyond 10 μM [2, 3]. At low concentrations, copper ion is an essential micronutrient that plays a variety of functions in biological systems. Copper containing enzymes and transcription factors are essential for cellular integrity, energy production, signalling, proliferation, oxidation and radiation defence. Research concerning acute or chronic toxicity of copper due to its deficiency or excess is growing rapidly and interest in the subject is pervasive [4–12]. Nevertheless, the pertinent redox status-dependent chelation [13–19] and regulatory mechanisms [20–32] are still being elucidated.

Recently, copper-related mechanisms have been suggested as therapeutic targets for important indications such as cancer [33], microbial defence [34–37], chronic lung inflammation [38], influenza A [39], neurodegenerative diseases including Alzheimer’s disease (AD), Parkinson’s disease (PD) and prion disease along with disorders linked to copper homeostasis such as Menkes disease (MD) or Wilson’s disease (WD) [40–43]. Elevated copper levels in the serum and tissue of cancer patients also suggest the involvement of copper in tumour growth [44, 45].

Our review will focus on biologically-relevant and emerging features of copper-dependent processes such as redox disproportionation, the properties of the chemical species generated (acid-base character, ligands, geometry etc. [46, 47]), the interaction between copper and sulfur redoxomes, the underlying redox signalling, along with the “dark side” where copper metabolism has been linked to compromised or fatal conditions [48–50].

### The redox capability of copper

**Fig. 1:**
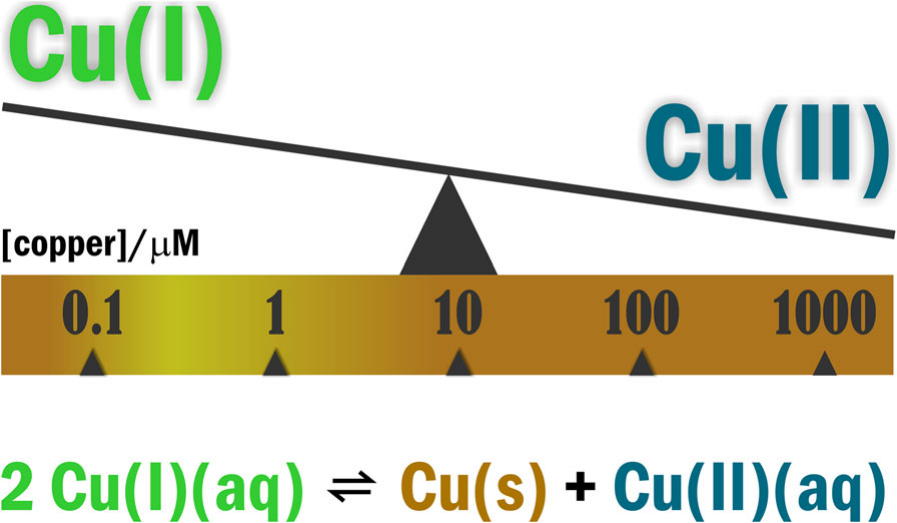
Disproportionation equilibria predicts Cu(I) in excess in the submicromolar to low micromolar range of ambient copper concentration. Due to the narrow non-toxic window for copper concentration, even small conditional changes may turn control into deregulation of copper signaling

Evidence for the incorporation of oxygen atoms from dioxygen (O_2_) into oxidation products of cuproenzyme-catalyzed reactions in nature was first published in 1955 [51]. Since the pioneering work of Osamu Hayaishi, and independently Howard S. Mason a consensus has been achieved as to the involvement of Cu(I) disproportionation (redox) equilibria 2Cu(I)(aq)ᅟCu(0)(s) + Cu(II)(aq) (**Eq. 1.**) in the aqueous reduction of O_2_ to water (*see* [52–54] and citations included). The value of + 0.37 *V* relating K=Cu(II)]/[Cu(I)]^2^ = 10^6^ *M*^− 1^ indicates that aerobic organisms can effectively utilize O_2_ when excess Cu(I) is sufficient [46]. This condition can be achieved within a copper concentration range of 10^− 7^ *M* to 10^− 6^ M (Fig. 1**.**) Within the 10^− 4^-10^− 3^ *M* range, however, the reduced Cu(I) form is minimally present, which would impair oxidative energy-transfer. The pertinent copper-containing enzymes such as cytochrome c oxidase (COX) [55] or copper, zinc superoxide dismutase (Cu, Zn-SOD1) [56–58] are involved in the mitochondrial electron transport chain [59] or in the dismutation of superoxide radical anion (O_2_^• -^) to hydrogen peroxide (H_2_O_2_), respectively. It is worth noting that the higher oxidation state of copper, Cu(III) may also shape the redox activation of the cytosolic copper pool and contributes to hydroxylation of phenolate substrates [60–62].

Cuprous Cu(I) ion possesses both electron “donor” and “acceptor” attributes, and redox capability via the one-electron transfer charge-disproportionation between the “donor” and “acceptor” Cu(I) yielding Cu(II) and Cu(0). This ability of Cu(I) to disproportionate is fundamental, not only to vital functions related to O_2_ transport, regulation of respiration, neuronal differentiation and signal transmission [63, 64], but also to the instability of the copper ionome [26]. We know that uncontrolled redox reactions of copper that can be deleterious to life [12, 65–75], however, here we focus on and re-consider the controlled, redox capability-related signalling of copper that may be important for neurobiology.

### Copper homeostasis

An evaluation of the effects of copper under normal and pathological conditions depends on an accurate knowledge of copper concentrations present in vivo. In spite of this, a bewildering feature of efforts to examine the role of copper in biological processes is the limited data available on the relative distribution of copper between organs, tissues, cell types and sub-cellular compartments in mammals [2, 44, 76–83]. From a practical viewpoint, the lack of the information makes it unrealistic to determine the recommended concentration of copper in drinking water. In addition to its biological variance, the significant differences in copper levels that exist in habitats and diets may also explain difficulties in determining the impact of copper on biological systems [2]. Moreover, multiple comparisons of existing data are compromised by the use of varying techniques, characteristically atomic absorption spectroscopy (AAS), flameless atomic absorption spectroscopic technique (FAAS), inductively coupled plasma-atomic emission spectrometry (ICP-AES) (Tables 1 and 2) and radiotracer detection or diverse sample preparing protocols. Data obtained by FAAS on brain tissue samples taken from 38 brain regions of 7 males within 2–4 h after death showing no macroscopic signs of disease [77, 78] disclosed significant copper concentration differences between brain areas, grey versus white matter cells, and between individuals. Brain copper concentrations were inversely correlated with age. It is worth noting that measurements of total copper levels may not necessarily reflect the biologically active metal pools [84].

**Table 1:** Average concentration of copper in human organs

	Sumino et al. [82]	Margalioth et al. [44]	Hamilton et al. [364]	Yoo et al. [83]	Lech & Sadlik [365]	Haswell [79]	Bárány et al. [76]
	FAAS	AAS	AAS	ICP-AES	FAAS	AAS	ICP-MS
	μg/g wet tissue						
brain	5.1			3.10	3.32		
liver	9.9		7.8	5.60	3.47		
kidney	2.6	1.80	2.1	1.80	2.15		
stomach		1.44			1.10		
intestines	2.1				1.54		
lung	1.3			0.97	1.91		
spleen	1.2			0.88	1.23		
heart	3.3			2.40	3.26		
bile					3.60		
blood^a^			1.2	0.97	0.85	0.99	0.95

^a^μg/ml fluid

**Table 2:** Average concentration of copper in different brain areas

	Bonilla 1984 [77]	Harrison et al. [78]	Ramos et al., [81]	Pal et al. [80]
	FAAS	AAS	ICP-MS	AAS
	μg/g dry tissue	μg/g dry tissue	μg/g dry tissue	μg/g wet tissue
Frontal pole	18.95			
Precentral gyrus	8.68			
Occipital pole	21.61			
Calcarine cortex	23.07			
Postcentral gyrus	18.83			
Supramarginal gyrus	16.45			
Uncus	16.30			
Cingulate gyrus	15.14		57	
Mammilay bodies	19.65			
Superior colliculus	15.38			
Inferior colliculus	17.92			
Olfactory tract	17.66			
Olfactory bulb	27.92			
Optic nerve	17.79			
Optic chiasm	7.06			
Caudate nucleus (head)	13.49	42	61	
Caudate nucleus (body)	18.46			
Caudate nucleus (tail)	23.12			
Putamen	14.62	44	62	
Globus pallidus	12.47	35	45	
Thalamus	8.75	21		
Frontal lobe, white matter	5.43	22	36	
Frontal lobe, gray matter		38		
Occipital lobe, white matter	8.88		55	
Parietal lobe, white matter	7.27		60	
Temporal lobe, white matter	11.12			
Red nucleus	10.41			
Substantia nigra	17.42			
Inferior olivary nucleus	12.00			
Superior olivary nucleus	17.46			
Pineal gland	17.81			
Cerebellum (vermal cortex, superior half)	10.92			
Cerebellum (vermal cortex, inferior half)	15.52			
Hippocampus		29	70	
Corpus callosum		14		
Cerebellum, gray matter		47	36	2.69
Cerebellum, white matter		22		
Frontal cortex			62	
Superior temporal gyrus			61	
Middle temporal gyrus			68	
Midbrain			38	
Pons			33	
Medulla			35	
Cortex				2.20
Striatum				2.18

Transition metals in biological tissues have been evaluated by atom absorption spectroscopy or radiotracer detection techniques, and more recently by the laser ablation inductively-coupled plasma mass spectrometry (LA-ICP-MS), secondary ion mass spectrometry, X-ray fluorescence microscopy (XFM), X-ray absorbance spectroscopy (XAS), micro particle-induced X-ray emission, and electron microscopy. Innovative imaging technologies of transitional metals were reviewed recently [85–90]. The recent development of recognition-based copper sensors and reaction-based copper indicators has allowed fluorescence imaging of labile copper pools [91–95]. Recent advances in non-destructive analytical methods will likely enable the assessment of copper dynamics over short, medium or long time scales that are relevant to signalling, metabolism and nutrition or aging.

These technologies have made possible a deeper understanding of copper dynamics and distribution. Significant relationships regarding the levels of Ctr1, Atox1, ATP7A/ATP7B and copper concentrations in the human brain have been identified by the combined application of ICP-MS spectrometry, Western blot and immunohistochemistry. Copper and ATP7A levels in the *substantia nigra* and in the *cerebellum*, respectively, have been found to be significantly greater compared to other brain regions [96]. New insights into the relative distribution of copper among elements including P, S, Cl, K, Ca, Fe, Zn within the *choroid plexus* (CP), ventricle system, and surrounding brain tissue have been provided by XFI techniques. In agreement with the known abundance of specific metal transporters, the elemental maps indicate that Zn, Fe and Cu are present within the CP, where the blood-cerebrospinal fluid barrier is primarily located [96–99]. Investigating the relationships between age, copper levels, and regulatory genes in the neurogenesis active sub-ventricular zone (SVZ) and CP has revealed i) age-related increases in Cu levels in both areas; ii) an age-related increase in MTs in SVZ, and iii) an age-related decrease and increase in Ctr1 in SVZ and CP, respectively [98]. These and past [100] findings suggest a specific role for copper in the development of brain tissue. The development of new imaging methods should provide a basis for further examination of the genuine labile copper pools, and related redox signalling within the brain.

### From atomic structure to Speciations shaping dynamic copper Pool and Signalling

Among transition metal elements in brain, copper ranks third only to iron and zinc in pervasiveness. Yet, its disproportionation chemistry is unique due to its electronic structure (3*s*^2^3*p*^6^3*d*^10^4*s*^1^) characterized by small energy differences between 3*d* and 4 *s* orbitals that allows for strong hybridization effects and electron tunneling [101, 102]. The easily convertible redox states Cu(I) and Cu(II) generate distinguishable bioligand variations (speciation). Indeed, axial symmetry distortion of Cu(II) aquo-complexes leads to extremely fast exchange of water (near to 10^10^*s*^*− 1*^) [103, 104]. This copper electron transfer-coupled structural alteration of coordination at copper sites in proteins [105, 106] can be envisaged as a molecular machine [107–109] switched on and driven by the redox disproportionation of copper. These molecular motions permit straight energy transfer from O_2_ to intrinsic cellular processes, potentially supporting fast neuronal signalling and remodelling of neuro-glia coupling [110] within the brain.

The extremely diverse copper speciation may be represented by a collection of copper bioligands including small ions and molecules such as sulfide ion, amino acids like His, Cys, Met, Asp, Tyr, Thr, Gly; neurotransmitters such as ATP, norepinephrine [111]; γ-aminobutyric acid (GABA) [112], and constituents of dense core vesicle cargo neurotrophins ([113] and references cited) inositol phosphates (IPs) [114], low-density lipoproteins (LDL) [115]. Redox propensity of chelates between copper and pertinent peptides (tripeptide glutathione (γ-L-glutamyl-L-cysteinylglycine: GSH) [116, 117]; peptide fragments of matricellular calcium-binding glycoprotein (secreted protein, acidic and rich in cysteine: SPARC) Gly-His-Lys (GHK) (for a recent review see [118] and proteins (metallothionein, ceruloplasmin, albumin, macroglobulin, transcuprein [3, 19, 119–122]), prion protein PrP^C^ [65], amylin [123]) may present specific feature of transport and storage of copper. Likewise, many cuproproteins with redox, or redox-with-transport functions (mono-, di-, tetranuclear cupredoxins nitrite reductase, laccase, Cu, Zn-SOD1, amine oxidase CuAO, galactose oxidase, hemocyanin, tyrosinase, catechol oxidase, COX, N_2_O reductase, menaquinol NO reductase et cetera) [47], copper-transporting ATPases (Cu-ATPases, ATP7A and ATP7B) [124–126], divalent metal transporter DMT1 [127], copper transporters and chaperons Ctr1, Ctr2, Atox1 and CCS [128, 129], diverse group of bacterial periplasmic copper binding proteins (CopC) [130] are known. It is to note, that major molecular players of growth or metabolism DNA [131] or biogenic polyamines (pAs) [132] also bind copper. It is to note, that the four metal binding sites of albumin are partially selective, transporting not only Cu(II) but also Zn(II), Ni(II), Cd(II), Pt(II), V(IV)O and Au(I) [133]. Besides, the rather unique redox stability of Cu(II) bound to the the *N*-terminal albumin sequence could also be explained by the presence of the axially coordinated water [133], presenting less-distorted pyramidal symmetry [103].

Through the application of multiple complementary approaches, two subsets of total copper can be distinguished: the static, tightly bound and the dynamic, relatively weakly bound (labile or exchangeable) pools [134]. Most of the copper uptake in cells takes place through the Ctr1, whereas ATP7A and ATP7B prevent excess copper accumulation within cells [125, 126]. The membrane protein Ctr1 is considered as the major entry pathway for copper into eukaryotic cells. Although it is currently the sole identified transporter for copper uptake, the existence of Ctr1-independent copper entry by as yet unknown transporters has been suggested [135, 136]. Copper entrance requires its prior reduction by cell surface metalloreductases, as Ctr1 mediates transport of Cu(I) only, whereas ceruloplasmin, which carries half of the copper in blood plasma, delivers it as Cu(II) to the cell membrane [137]. Copper uptake is regulated mainly by Ctr1 translocation between the membrane surface and intracellular vesicles on demand, however, the Ctr1 protein has been shown to be degraded more rapidly under conditions of high copper excess [136].

Binding events in the His- and Met-rich extracellular amino terminal domain of vertebrate Ctr1 may support both reduction and transfer of copper from the carriers to the transporter [138]. Questions arise how Ctr1- bound copper moves outside-in down the peptide chain and dissociates? The human transporter is a symmetric Ctr1 trimer shaping a cone-like pore, which becomes wider in the outside-in direction from approximately 8 *Å* to 22 *Å* [129]. Cu(I) may traverse from the extracellular binding site through the cone to the HisCysHis motif near the intracellular carboxyl terminus of the protein by exchanging neighbouring Met of the conserved Met-XXX-Met Cu-binding motifs positioned along the pore interface. Higher stability of Cu(I)-Cys versus Cu(I)-Met could be the driving force for Cu(I) passage [136]. As far as the intracellular Cu(I) discharge pathways are concerned, Cys containing small peptides, such as GSH, or the “antioxidant peptide” Atox1 may contribute to Cu(I) release from the carrier. The typically high intracellular concentration of GSH (*cca*. 10 mM [139]) may produce shifting of the binding equilibrium towards GSH bound Cu(I) suggesting that GSH can efficiently collect copper [140, 141] bound to the intracellular HisCysHis binding crevice of Ctr1. Alternatively, Atox1 can also pick up HisCysHis bound Cu(I) and shuttles it to cytoplasmic metal-binding domains in ATP7A and ATP7B (also called MD and WD proteins, respectively) [16, 63, 142–144]. As suggested previously, the fast exchange of amino acid residues surrounding Cu(I) can readily explain entropy-compensation phenomena in course of dynamic interconversion of Cu-Cys coordinations during chaperon-target hopping [144].

**Fig. 2:**
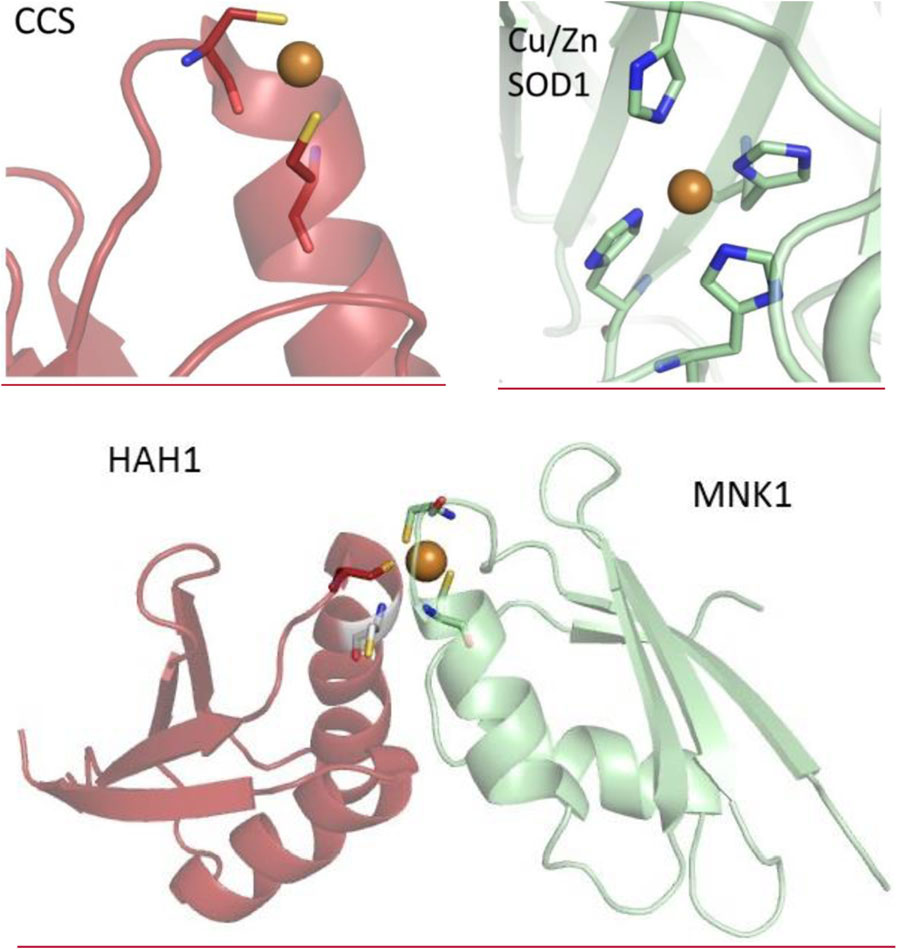
Diverse speciation of copper in chaperons and targets. Upper row left: The two Cys residues Cys22 and Cys25 of the first domain of CCS chaperone (PDB code: 2rsq) [149] bind copper (yellow) with an average distance of 2.2 *Å*. Upper panel right: Copper (yellow) delivered to the target enzyme Cu, Zn-SOD1 (PDB code: 2C9V) [150] is bound by four His residues His46, His48, His63 and His120, and characterized by a range of Cu-His distances from 2.1 *Å* to 2.5 *Å*. Lower panel: The position of copper in the chaperon-Cu-target complex between chaperon HAH1 (magenta) and the first domain of the target ATP7A (Menkes protein, MNK1) (green) (PDB code: 2k1r) [152]). Three Cys residues fitting in both HAH1 (Cys12, Cys15) and MNK1 (Cys15, Cys18) CXXC motifs participate in the transition of copper from HAH1 to MNK1 [152]. Specifically, Cys12 of HAH1 and Cys15 of MNK1 are required for the formation of the HAH1-Cu-MNK1 complex, while the third Cys may be either of the Cys15 of HAH1 or the Cys18 of MNK1. Three coordinating Cys side chains are shown around the copper ion, all with a distance of 2.1 *Å*, the fourth Cys, which does not bind the metal thus far, is shown in green

The astonishing fact that free copper is undetectable within cells is due to the existence of copper chaperones, such as CCS which binds and transfers copper directly to its final target Cu, Zn-SOD1 [145, 146]. One can assume a novel type of protein-protein interaction delivering copper to its protein target destinations intracellularly [147]. It has been suggested that the exchange of copper between a variety of target-specific cytosolic chaperones and their targets in distinct compartments is driven by an increase in the copper binding affinity [148]. The speciation of copper sites in the CCS chaperone for target Cu, Zn-SOD1 and in the HAH1 chaperone for the soluble cytosolic domains (Menkes protein, MNK1) of the target ATP7A (Fig. 2**.**) [148–151] indicates, that the first domain of CCS, the sixth domain of the MNK1 and the HAH1 binds Cu(I) through two cysteines in a Cys-XX-Cys motif. The Cu, Zn-SOD1 protein binds copper via four His residues. Reportedly, the values of the apparent dissociation constants of Cu(I) towards chaperones and their intracellular targets may vary mostly in the range of 0.01*pM* to 0.1*fM* [148]. These estimates, however, turned to be erroneous as demonstrated by Shoshan and co-workers [152]. By taking into consideration oligomeric species of Cu(I)-dithiothreitol, the modified calculations conclude affinity values several orders of magnitude higher, an observation that deserves further comments. The affinities of copper sensors and indicators associated with novel imaging technologies may not allow fluorescence imaging of strongly bound copper in chaperones or targets, but possibly will permit detection of chaperone-Cu(I)-target complex formation (Fig. 2**.** lower panel), as characterized by several orders of magnitude lower affinity for the complex formation equilibria MNK1 + HAH1MNK1-Cu(I)-HAH1 (**Eq. 2.**) [151]. In this case, variations of AAS (total pool) and fluorescence imaging (labile pool) data could give rise to proper assess of the strongly bound copper pool.

### Intracellular cu(I), GSH and the concept of coupled “Redoxomes”

As outlined above, intracellular copper exists in an immense variety of static forms that involve a multiple oxidation states with favoured ligand speciations (*see* below) or mixed-valent copper complexes. However, it also can change by reacting with “self” (*see* **Eq. 1.**) within sub-nanometer distances of multiple copper sites of vital peptides, proteins and enzymes [101, 102, 105, 106, 153–156]. A somewhat similar distinction can be made for a sulfur “redoxome”, a redox reaction-coupled proteomic network comprising numerous sulfur oxidation states and species and reactions with sulfur-containing peptides, proteins and enzymes, as well as the reaction of GSH with “self” yielding glutathione disulfide (GSSG) (2GSH → GSSG) [157–159]. Importantly, these “self”-reacting copper and sulfur “redoxomes” also interact with each other through the prominent chelation of Cu(I) by thiols of either the antioxidant copper chaperone protein Atox1 or GSH [63, 142, 160–162]. Supporting this concept, the GSH/GSSG ratio was found to be the most sensitive indicator of copper intoxication (and subsequent oxidative stress) [11]. Moreover, sulfur-doped copper clusters are relatively stable and abundant [163]. In the Cu-S-Cu unit found within active sites of copper-sulfur proteins like COX, the S^−^ bridging a Cu_2_^+^ component displays a short Cu-Cu distance and a small Cu-S-Cu bond angle, which are essential for the electron transport performed by COX [163–165]. With this in mind, toxicity of copper excess in mammalian cells is explained by obstructing the control of the “interactome” of copper-sulfur containing “redoxomes” [166–169].

Because of its charge density and polarizability, the oxidized cupric Cu(II) ion would tend to be found in complex with “hard” bases such as H_2_O, OH^−^, RNH_2_ etc. (N- or O-ligands), while the “soft” acid Cu(I) does favour “soft” bases such as RS^−^ and CN^−^ ligands [46]. These trends in the stability of coordination complexes [170, 171] predict that the reduced cuprous Cu(I) ion would prefer the formation of complexes with “S-ligands” such as GSH [172]. Importantly, GSH can also represent “N- or O-ligands” for Cu(II), assisting disproportionation and O_2_^• −^ dismutase activity of Cu(I). Indeed, the complex equilibrium system GSH-Cu(I) can switch to the oxidized GSSG-Cu(II) one [173]. Taken together, these observations have been used to classify the speciation of Cu(I) with GSH as a key feature accompanying redox homeostasis [11].

Although the dissociation constant for the GSH-Cu(I) equilibrium has been predicted to be about 9*pM* [148] (GSH-Cu(I)), this value has been called into question [174, 175]. Specifically, in an experimental model of GSH-Cu(I), the formation of the tetranuclear [Cu_4_(GS)_6_] cluster was observed as the major species within the range of pH from 5.5 to 7.5 [175]. The cluster formation equilibrium predicts that [Cu_4_(GS)_6_] limits free Cu(I)(aq) to the sub-femtomolar concentration range in eukaryotes. These findings suggest that the affinity of GSH towards Cu(I) may be orders of magnitude higher than previously thought [148]. If valid in vivo, not only the high intracellular GSH concentration but the high-affinity formation of the [Cu_4_(GS)_6_] cluster would also force the membrane-cytosol transfer of Cu(I) from Ctr1 to GSH. It is noteworthy, that bacteria capture copper surplus through the cytosolic protein Csp3s, which forms tetranuclear Cu(I) thiolate clusters [176] [Cu_4_(S-Cys)_5_]^−^, [Cu_4_(S-Cys)_6_]^2−^, and [Cu_4_(S-Cys)_5_(O-Asn)]^−^. In order to avoid toxicity of cytosolic copper overload, eukaryotes gain control over excess by MTs [177], including the brain-specific MT3 (growth inhibitory factor) binding. In fact, using XRF microscopy with sub-micron resolution, Sullivan et al. [178, 180] demonstrated the presence of Cu-rich aggregates in astrocytes of the dentate gyrus and rostral migratory stream in the rat brain. These aggregates contain Cu_x_S_y_ clusters with a sulfur/Cu(I) ratio consistent with that of the Cu-MT complex. Apparently, both age-dependent [98] and overload-evoked changes [177–180] can be related to the copper-binding capacity of MTs.

Direct and indirect effects leading to sudden and catastrophic hemolytic anemia due to the direct toxic effects of copper on red blood cells has been described in the past [181–190]. Nevertheless, the observation that during chronic copper poisoning in sheep there is decreased antioxidant capacity directly correlating with the level of serum copper [191] putting GSH at the centre of anti-ROS protection [192]. Underscoring the importance of this role, the level of GSH in erythrocytes is an inheritable trait [193]. Unfortunately, it is hard to obtain valid GSH and GSH/GSSG data from biological samples [194].

### Central regulation and storage of copper: Copper deficiency and toxicity disorders

ATP7A and ATP7B are highly abundant in the liver, yet disruptions in their transport functions affect the central nervous system (CNS). This is reflected in the sex-linked recessive CNS disorder observed in males with symptoms of copper deficiency (MD) arising from a mutant ATP7A pump. In contrast, a mutant ATP7B pump leads to copper toxicity in the autosomal recessive WD. These “brain” diseases suggest that the homeostasis of copper in the liver is essential for normal brain function [195–197]. It has been known for a long time, that WD is characterized by the accumulation of copper in tissues, particularly in the liver and brain ([198–201] and references cited). The biosynthesis, folding, localization, turnover and protein interaction network, of the most frequent copper transporter ATP7B mutant causing toxic accumulation of copper in WD has recently been described [202]. By targeting this network with specific siRNAs, correction of the localization of ATP7B-mutant restored copper levels to an acceptable range. Decreased stability associated with increased structural dynamics has been ascribed to disease-causing point-mutations in the metal-binding domains of WD protein [203]. Another fatal liver injury, the Indian childhood cirrhosis (ICC), was also found to be associated with heavy deposits of copper, though in all other respects it was different from WD [204].

Besides their significance in the overall copper efflux and balance, ATP7A and ATP7B play a critical role in copper transport between intracellular compartments. In hepatocytes, ATP7A and ATP7B are located mainly in the trans-Golgi network and supply copper for incorporation into copper-dependent enzymes such as tyrosinase, peptidylglycine amidating monooxygenase, dopamine monooxygenase, lysyl oxidase, and ceruloplasmin [205]. At high intracellular copper concentration, the carriers are translocated reversibly to the plasma membrane (ATP7A typically to the basolateral, ATP7B to the apical surface) where they efflux excess copper from the cell [206].

In food and water, the average daily intake of copper in the US is about 1 mg [207], which is relatively low. Most humans and animals are able to control excess amounts of copper by either decreased absorption or increased excretion. Ingestion of toxic amount of copper (> 10 *mg/day*) or acute or chronic environmental exposure, such as occupational hazard, accidents, release from copper pipes, initially affects the liver, the first organ of copper deposit. Many factors that alter copper metabolism influence the progress of chronic copper poisoning. The toxicity remains subclinical until the copper that is stored in the liver is released in massive amounts. The lethal dose of copper is about 10-20 *g* [207]. Initial symptoms of acute overdoses may be metallic taste, gastrointestinal distress that can progress to cardiovascular collapse, coma and death within hours. Hepatic symptoms arise after 24 *h* to 72 *h* of exposure, and are characterized by marked elevations in serum aminotransferase levels, hepatic failure, elevation in prothrombin time and jaundice. Erosion of epithelial lining of the gastrointestinal tract, acute tubular necrosis in the kidney was also reported. Blood copper concentrations can increase suddenly, causing lipid peroxidation and intravascular hemolysis [207, 208].

The liver takes up dietary copper from the portal blood, synthesizes cuproproteins in hepatocytes, and secretes excess copper into the bile. Overall balance of copper in the body is achieved by regulation of the rate of uptake in the small intestine and of biliary excretion. The key regulators of these processes are the ATP7A and ATP7B pumps. However, many other components of the machinery for copper homeostasis have been described including ceruloplasmin, small carriers, chaperones, MTs [24, 197, 205, 209, 210]. Precise regulation of intracellular copper homeostasis is essential, which is supported by the large number of clinical syndromes linked to either copper excess or shortage [197, 210, 211]. Several reviews have summarized results of genetic, biochemical and structural approaches concerning cellular copper homeostasis and related disorders [116, 212, 213], yet the entire network of events that regulate copper transport and intracellular disposition has not been fully explored.

As Ctr1 cannot transport bivalent copper, some ingested Cu(II) avoids the liver and passes rapidly into the systemic circulation where can target albumin [135]. Following entry into hepatocytes, Cu(I) binds the initial acceptor GSH, which delivers it to the different copper chaperones, such as Atox1, CCS and COX17 that partition copper into distinct intracellular compartments [116]. Nevertheless, the landscape of Cu(I) trafficking to chaperons via GSH may change. Recent data on femtomolar [175] versus picomolar [148] affinities of GSH towards Cu(I) raise the role for [Cu_4_(GS)_6_] preserving Cu(I). In fact, the Cu(I) availability is highly associated with GSH level of the cell. Ogra et al. [214] observed that depletion of GSH led to decreased copper in the bile and blood but increased copper in the liver. The decreased GSH level resulted in an oxidative environment in the liver that made Cu(I) less bioavailable. In addition, the redox state of the cells influences the activity of copper pumps. GSH deficiency inhibits ATP7A and ATP7B resulting in the intracellular accumulation of copper [136].

### Synaptic release of copper

The concentration of copper in the cerebrospinal fluid (~ 70-80 μM) is rather high in comparison to serum (12-24 *μM*) [215, 216], raising the possibility of specific copper signalling in the brain. As outlined in previous sections, most cellular copper is strongly bound to proteins, yet the disposition of loosely bound copper can be detected by novel imaging technologies. This labile copper pool is believed to be associated with redox signalling. Labile copper has been found in the soma of cerebellar granule and cortical pyramidal neurons, in addition to the neuropil in the cerebellar and cerebral cortices, hippocampus and spinal cord [217].

The observation on the release of zinc from brain tissue during activity published in Nature in 1984 [218] provided initial evidence that transition metals could be directly involved in signalling [112, 134, 219–229]. Initial evidence suggested the potential of copper to modulate brain activity by affecting central inhibition. These include findings such as the pro-convulsant effects of a hitherto unidentified endogenous substance containing copper [112, 230], or depolarization-induced co-release of endogenous copper with the major inhibitory neurotransmitter γ-aminobutyric acid (GABA) in different experimental models of nerve terminals in vitro (synaptosomal fraction) and ex vivo (*median eminence*) [225]. Conclusions from ^67^Cu uptake and release measurements performed in hypothalamic slices by the presence of action potential blocker tetrodotoxin [231, 232] or determination of depolarization-induced copper release from nerve endings by AAS [225] raised the concept of copper signalling in the brain. Findings, such as the N-methyl-*D*-aspartate (NMDA) receptor activation-induced ATP7A trafficking to the plasmamembrane in the hippocampus [233, 234] have have provided new support for a role for copper efflux in mechanisms of excitotoxicity.

**Fig. 3:**
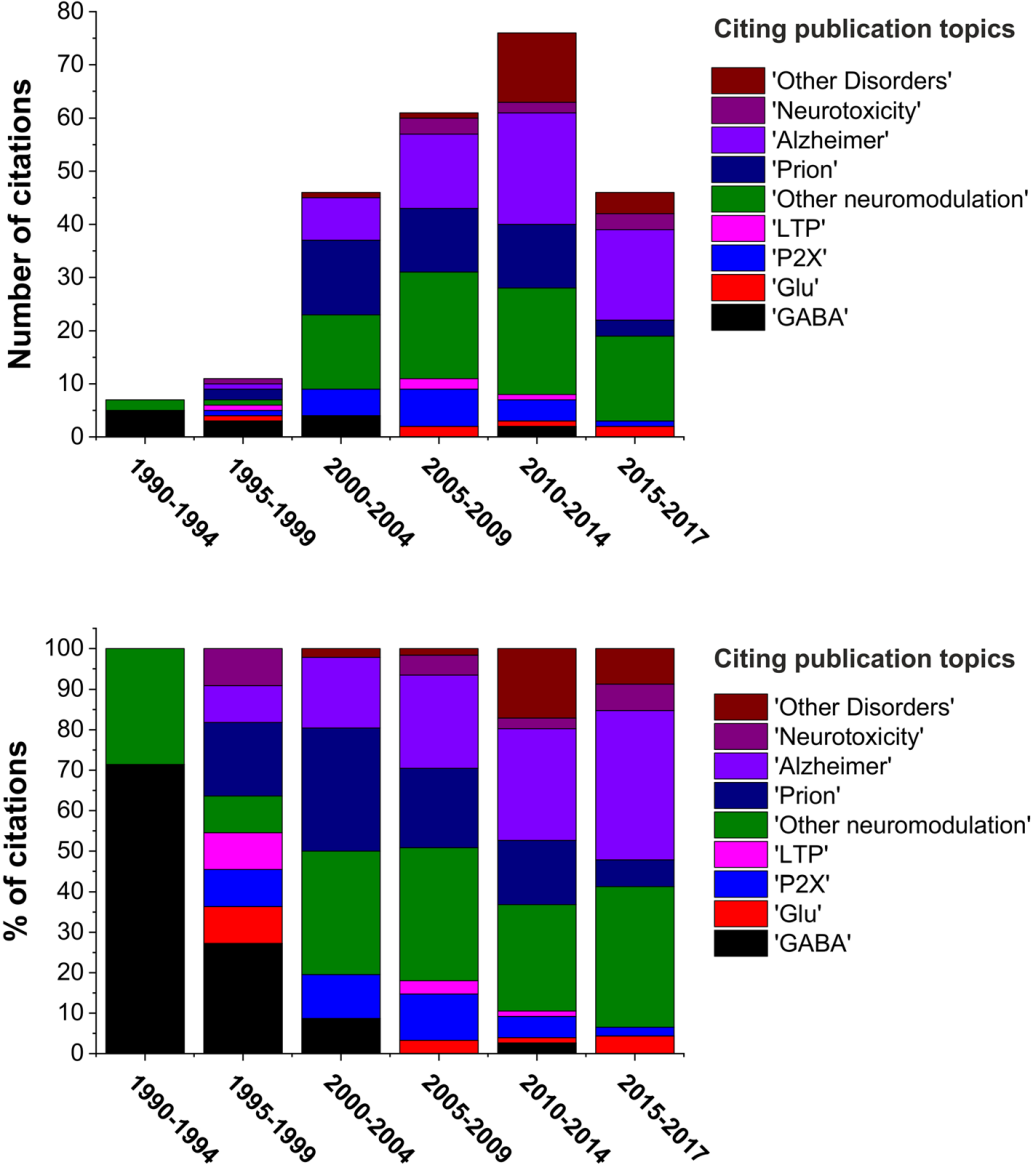
Emerging themes of copper signalling and functions. Number (Left) and percentage (Right) of papers citing the first description of depolarization-induced synaptic copper release [225] in each subject category by 5-year intervals. From the time, copper signalling in brain have considerably been developed, including inhibitory and excitatory signalling, neuromodulation, neurotoxicity, Alzheimer’s and other brain disorders

During the past 30 years, there has been a renaissance of interest and an expanded view of the contributions of copper to brain function and pathophysiology, as reflected in follow-up statistics, and throughout the literature [18, 42, 63, 69, 235–248] (Fig. 3**.**). One may speculate about copper speciation and/or mechanisms of copper uptake and release. Apart from trafficking in complex with various neurotransmitters, carrier peptides and proteins, or as part of the protein cargo of extracellular vesicles [39] there is also the potential for copper uptake as a result of autophagy [29, 249].

Zinc released from brain tissue during activity has been shown to reach concentrations in the hundred micromolar range, e.g. 300 μM [218]. In contrast, the concentration of copper in the synaptic cleft has been claimed to range from 1 to 10 *μM,* as determined by using the fluorescent indicator tetrakis-(4-sulfophenyl)-porphine (TSPP) in bovine chromaffin cells [250]. Notwithstanding the importance of imaging heavy metals in vivo (*see* also section 2.1. “*Imaging technologies*”), the quantitative relevance of fluorescent indicators strongly depends on the affinity standards applied (*see* for example [148]). Furthermore, TSPP has several drawbacks that can limit the validity of data obtained: *i*) the sub-micromolar Kd value of the TSPP-copper complex may not provide accurate data at the point of/or above saturation; *ii*) TSPP-copper binding is influenced by dissociation of copper from protein binding sites which necessary to validate the data an approach that is independent of protein binding, as in the case of ICP-MS technique; and finally, *iii*) the weak fluorescence intensitiy of the TSPP ligand itself. Conversely, based on atomic absorption spectroscopy data on depolarization-induced release of copper from nerve endings, one can estimate an activity-dependent enhancement of copper in the synaptic cleft, in the range of 100-250 μM [225], depending on the cleft size and volume taken. Furthermore, based on the Kd (100 *μM*) and saturating GABA concentration (1 *mM*) for GABAa receptor binding and desensitization [251–254] and assuming a stoichiometry of 2 for GABA co-released with copper [112] one could also conlude that a copper concentration of 100 *μM* can exist transiently within the synaptic cleft.

Copper can diffuse out of the synapse driven by the lower extrasynaptic concentration (1 *μM*) [216]. Moreover, the extrasynaptic copper concentration has been estimated to be in the nanomolar range based on the cellular and network excitability produced by bath-applied copper in the CA1 area of the rat hippocampal slice [244]. (This effect was primarily explained by the ability of copper to interfere with Hodgkin–Huxley conductances rather than the synaptic effects of copper [255].) Using a second-generation fluorescent copper sensor in combination with XFM, Dodani et al. [256] have observed that neural activity triggers copper trafficking from the cell body toward dendrites and revealed that these copper fluxes are calcium-dependent. This work provided direct imaging evidence that complemented prior studies on bulk copper release [225, 232]. Applying fluorescent copper indicators with improved hydrophilicity Dodani et al. [256] identified labile copper sources in the developing retina, and demonstrated that they modulate spontaneous activity of neural circuits via the copper transporter Ctr1, referred to as a ‘copper ion channel’ (*see* also section “*The source-target-physiology scheme for therapeutic intervention*”).

### Copper Dyshomeostasis and brain disorders

Chronic copper intoxication causes region-specific copper accumulation in the CNS of male Wistar rats, following intra-peritoneal injections of copper lactate (0.15 *mg* Cu/100 *g* body weight) daily for 90 days. In these animals, copper content, but not that of zinc or iron was found to be significantly elevated in the cortex, cerebellum and striatum as determined by atomic absorption spectrophotometry [257]. Remarkably, metal dis-homeostasis has been widely accepted as a hallmark of several neurodegenerative diseases, such as prion, AD, PD, amyotrophic lateral sclerosis (ALS), Huntington’s chorea (HC) ([257, 258] and references cited). The antioxidant responses to copper overloads (0–30 *mg/kg*) in rat brains showed markedly decreased brain GSH and GSH/GSSG ratio after chronic copper exposure. Copper overloads are characterized by a t_1/2_ of 9-10 *h* for the decrease in GSH and of 4 *h* for decreases in the GSH/GSSG ratio, the latter being the most sensitive indicator of copper excess [11].

#### Prion diseases

The mainly α-helical folded prion protein PrP^C^ is expressed in the enteric nervous system, e.g. in enteric nerve fibers/terminals and glia within the myenteric submucosal plexuses (inguinea pigs, mice), suggesting a role in the regulation of ileal contractility [259]. Additional beneficial roles for PrP^C^ may arise from the discovery, that prion is an agonist at the G-protein coupled Adgrg6 receptor, known to regulate demyelinization-linked neuropathy [260, 261]. Copper has long been associated with the formation of protease-resistant, β-sheet enriched “scrapie” conformation of prion protein PrP^Sc^, which has been considered the critical step in the neurodegenerative prion diseases known as transmissible spongiform encephalopathies [43, 262]. Recently, Giachin et al. [263] proposed that there is a non-octarepeat copper binding region [264] of PrP^C^ which switch to the infectious PrP^Sc^ under acidic conditions. The only known prion disease observed in wildlife is the chronic wasting disease (CWD). Dietary magnesium and copper have been linked to inflammatory events in CWD pathogenesis [265]. Importantly, geographical regions where CWD is absent have significantly higher concentration of magnesium, and region where CDW is endemic show a higher magnesium/copper ratio in the water. Prion diseases share characteristics of “prion-like” neurodegenerative diseases in terms of the involvement of proteins (α-synuclein, amyloid β, and tau) forming amyloid deposits [266].

#### Alzheimer’s disease

The metal theory of AD [43, 267–273] (but *see* the advice of Schrag et al. [274]) predicts that the disregulation of copper/zinc levels by proteins known to be involved in AD-related neurodegeneration may lead to the accumulation of amyloid fibers and oxidative stress. Indeed, by using XFM high areal concentration of copper has been detected in amyloid beta (Aβ) plaques of the hippocampal *gyrus dentatus* sub-region in a mouse model of AD [275]. These data corroborate previous findings on the high-affinity interaction between Cu(II) and the histidine binding motif of Aβ [276], along with the role for Aβ as a synaptic Cu(II) scavenger [277]. In addition, the experimental ‘halo’ effect in copper maps may indicate co-localization of copper with a ‘ring’ rich in lipids, observed around the Aβ plaque in AD models [278] and human AD sections [279]. This suggests a potential association between Cu-catalyzed oxidative stress and plaque formation [280]. However, the question remains as to whether changes in metal distribution are the cause or the consequence of the plaque formation and progression of AD [275] or other progressive neurodegenerative diseases. For example, the neuropathology seen in AD may also characterize individuals with Down syndrome [281, 282], ALS or HC. By supporting a common pathway for familial and sporadic ALS, the pathological inclusions containing SOD1 fibrils may hold amyloid-like properties [283]. Abnormal copper accumulation in the striatum of HC patients has been linked to the copper binding facilitated formation of amyloid- copper transporter Ctr1, rlike bodies of the huntingtin (Htt) protein [284, 285]. Differential effects of ATP7A and ATP7B regulating copper metabolism MURR1 domain protein 1 (COMMD1) on the formation of mutant Cu, Zn-SOD1 fibrils (increase) or parkin inclusions (decrease) as well as the Htt aggregates (unaltered), however, suggest mechanistic diversity [286].

#### Parkinson’s disease

There is evidence that alterations in copper homeostasis play a role in PD with excess copper leading to neuronal cell death and α-synuclein aggregation [121, 287]. It is noteworthy in this context, that the depletion of GSH [70] is a very early symptom in the course of PD [288]. Amyloid fibre formation in type-2 diabetes [289] may also facilitate PD, due to the acceleration of α-synuclein amyloid formation by islet amyloid polypeptide amylin [290]. Disruption of retromer, a conserved heterotrimeric protein complex consisting of VPS35, VPS29 and VPS26, has been observed in a number of diseases including PD [291], resulting in disregulation of the retrieval and recycling of vital proteins [292]. Furthermore, the mutation of VPS35 increases copper toxicity in yeast, a likely outcome of the copper transporter miss-trafficking [293]. In fact, the endosomal retrieval and recycling of the copper transporter ATP7A is retromer-dependent in human cells [294]. Protecting the cargo of regulatory membrane proteins such as copper transporters and pumps via the retromer shipment may be critical in age-related health. It will be important to consider further the link between retromer complexes and copper homeostasis.

#### Multiple sclerosis

Disease-specific autoantibodies against inwardly rectifying K^+^ ion channel 4.1 (Kir4.1) [295], have been identified in the sera of patients suffering from the chronic inflammatory CNS disorder multiple sclerosis (MS). Feeding the copper chelator bis-cyclohexanone-oxalyldihydrazone (cuprizone, CTZ) reduces the myelin sheath and activates microglial and astroglial cells in the CNS, providing a reproducible and reversible model of pathologic processes underlying white and gray matter demyelination [296–301]. Expression of Kir4.1 autoantigen has been studied in the brain of CTZ-fed mice and revealed the induction of Kir4.1 protein in microvessels of the cerebral cortex [302]. The antioxidant functions of MTs [303] may have a role in MS, as suggested by the elevated level of MTs induced by CTZ in astroglia, while the oligodendroglia express low levels of MTs, which may contribute to their oxidative stress vulnerability [304, 305]. Apart from MS modelling, several lines of evidence suggest that CTZ intoxication is an excellent paradigm to study pathology and/or therapy of epilepsy or schizophrenia as well. However, mechanistic clues claiming either copper deficiency or copper build-up (associated with hydrazide formation-dependent enzyme inhibition) remain contradictory [306].

### Chelate therapy

The restoration of copper homeostasis is mostly relevant to WD [119, 307], although neurodegenerative ([308–312], but see [313] versus [314]) or inflammatory [38, 208] diseases can also be related. Before the disease progresses to liver and brain (WD) or lung (inflammation), the excess copper can be limited by Cu(II) reduction, Zn(II) addition and administration of Cu(II) chelating ligands such as tetrathiomolybdate (TM), triethylene tetramine (Trientine, TETA, Trien) or D-penicillamine (D-pen) [119, 207] for limiting excess copper.

Due to its high level in proliferating tissues, copper can also promote angiogenesis and cancer development [315]. Hence, the Cu(II) lowering therapy has also potential in treating cancer (breast, colorectal, leukemia, lung, prostate) by copper chelating compounds (Table 3). A range of targets and/or mechanisms of action have been suggested for the antiproliferative activity of the Cu(II) chelate forming compounds. These include proteasome inhibitors and apoptosis inducers [316], DNA and protein interactions [317, 318], ROS generation [319], oxidative stress [320], integrin β4 up-regulation [321], Schiff base copper complex formation [318, 322]. (For a comprehensive knowledge of copper ion complexes as anticancer agents we refer to reviews [323, 324]).

**Table 3:** Copper chelating compounds with anticancer activities

Compound type (name)	Structure	Chemical name	Ligand type	Application
TM		Ammonium tetra-thiomolybdate Bis-Choline tetrathio molybdate	Bi-dentate	Breast, prostate, kidney cancer cells [208]
Trientine (TETA, Trien)		N,N′-Bis(2-aminoethyl) ethane-1,2-diamine	Tetra-dentate	Colorectal cancer cells [366]
Hydroxyquinoline (Clioquinol)		5-Chloro-7-iodo-8-hydroxy quinoline	Bi-dentate	AD and human breast cancer cells [309, 316]
D-pen		3-Mercapto-D-valine	Bi-dentate	Human leukemia and breast cancer cells [319]
Captopril		D-3-Mercapto-2-methyl-propionyl-L-proline	–	Mammary ductal carcinoma cell line [367, 368]
Dithiocarbamates				
Disulfiram (DSF, Antabuse)		1-(Diethylthio-carbamoyl-disulfanyl)-N,N-diethyl-methane-thioamide	–	Human breast, lung cancer cells [315, 369]
Pyrrrolidine dithiocarbamate (PDTC)		Pyrrolidine-1-carbodithioic acid	Bi-dentate	Human breast cancer cells [344]
Thiosemicarbazone				
Hydroxyquinoline-carboxaldehyde–Thiosemi-carbazone		8-Hydroxy-quinoline-2-carbox-aldehyde–thio-semicarbazone	R = H tetra-dentate	Prostate cancer cells [370]
Retinal thiosemicarbazone		9-*cis*-Retinal thiosemi-carbazone	Bi-dentate	Human leukemic cell U937 [317]
1,2-Bis(thiosemi-carbazones)		H_2_gts: glyoxal-bis(thiosemi-carbazone) atsm: diacetyl-bis(4-methylthio-semi-carbazone) ptsm: pyruvaldehyde-bis(4-methylthio-semicarbazone)	Tetra-dentate	atsm: human colon cancer tumor cells ptsm: Ehrlich ascites and EMT6 tumour cells [371]
Elesclomol		N’^1^,N’^3^-Dimethyl-N’^1^,N’^3^-bis(phenyl-carbonothioyl)propanedihydrazide	Tetra-dentate	Metastatic melanoma cells [320, 331]
Schiff-bases				
Salicylaldehyde-benzoylhydrazone (SBH)		N′-[(2-Hydroxyphenyl) methylidene] benzohydrazide	Bi-dentate [372]	Human adeno-carcinoma cell line [373]
Salicylaldehyde-pyrazole-hydrazone (SPH)		(*E*)- N′-(2-Hydroxy-benzylidene)-1-benzyl-3-phenyl-1H-pyrazole-5-carbohydrazide	–	Lung carcinoma cells [321]
Pyridine-carboxaldehyde-phenylpyrimidyl-hydrazone (Pyimpy)		1-Phenyl-1-(pyridin-2-yl)-2-(pyridin-2-ylmethylene)hydrazine	Tri-dentate	Rat breast tumor cells [322]
Hydroxy naphthaldehyde imine (HL)		1-(((2-((2-Hydroxy-propyl)amino) ethyl)imino) methyl) naphthalene-2-ol)	Tri-dentate	Human cervical and liver hepatocellular carcinoma cells [318]

Considering the redox activity of potential anticancer Cu(II) chelates (Table 3 and references cited) [323–327] one possible consequence is that the high level of copper in proliferating tissue could also reduce oxidative redox potential which may in turn increase cancer cell proliferation [45, 50, 328, 329]. The redox imbalance could be targeted by chelate formation coupled Cu(II) reduction in the proliferating tissue. Indeed, the reversible one-electron reduction of Cu(II) does occur, as exemplified by the thiosemicarbazone complex of Cu(II) in the elesclomol [330, 331] (Table 3).

It is interesting to note the effect of metformin [50, 332–334], which is a first line diabetes II drug and has been shown to increase healthy life span irrespective of its anti-diabetes effect. Its copper chelating ability [335] may suggest an anti-aging role for copper.

### The source-target-physiology scheme for therapeutic intervention

The advent of imaging techniques that gained insight into the dynamics of labile copper pool made it possible to look beyond the molecular-level interactions in copper homeostasis and examine network-level dynamic interplays shaping copper signalling. The source-target-nphysiology (STP) scheme suggested by Chang [336] includes labile, neuronal copper pools in the Golgi compartment as a source, signal propagation via postsynaptic membrane receptor/ion channel target (the Cu(I) transporter Ctr1), and copper-dependent spontaneous activity of the neural network (physiology). Vesicular storage of canonical neurotransmitters with copper suggesting their co-release has also been described. Furthering the interactions between compartments within neurons, we conceive that cellular-level copper signalling between neurons and astrocytes, an emerging cell type of the brain, also exists and may play a fundamental part in the brain’s information processing.

**Fig. 4:**
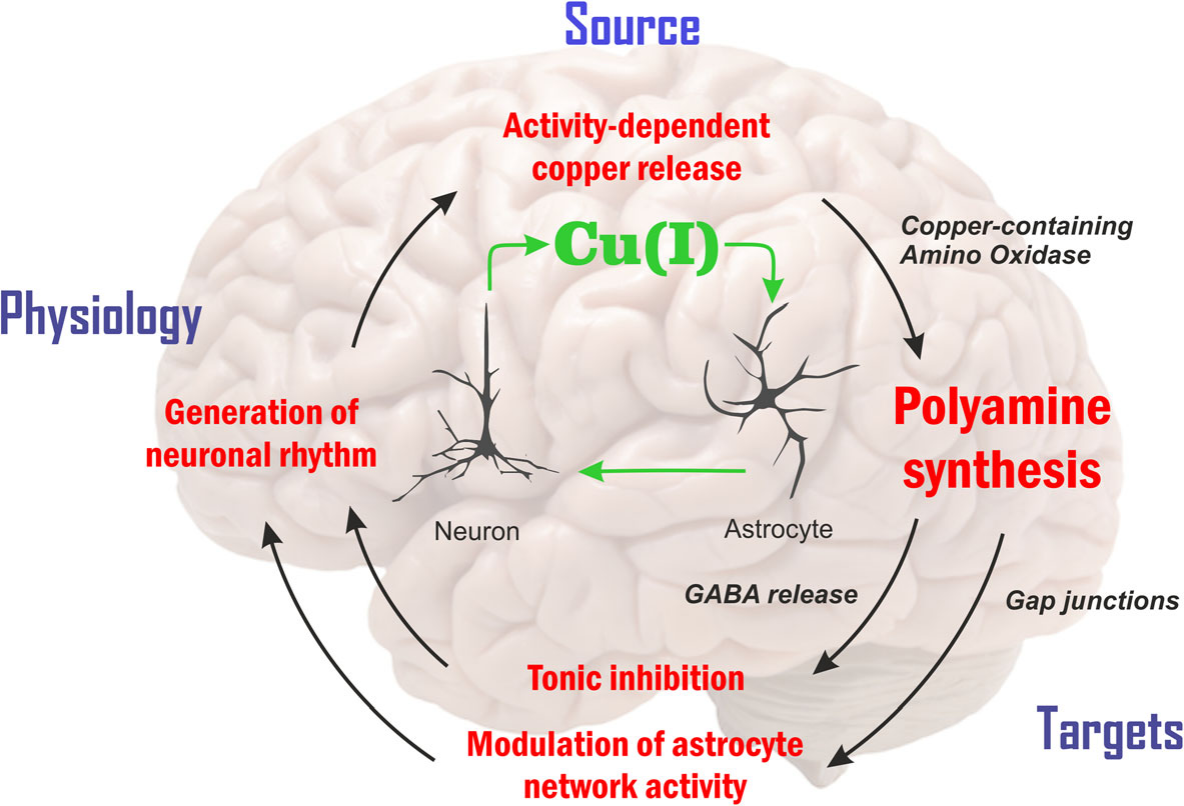
Copper signaling via neuro-glia coupling. Astroglia, a previously neglected cell type of the brain [340], operate a variety of copper-dependent metabolic functions [6, 80, 240, 341, 342]. For this reason, in addition to synaptic and extrasynaptic copper signalling by way of excitatory/inhibitory receptors and ionic channels [22, 234, 235, 237–244, 246, 255, 336, 345–355], we place copper-dependent production of pAs in astrocytes [338] and correlated gap-junction modulation in the centre of this option. The proposed scheme conjectures activity-dependent changes of copper pools [179, 180] and polyamines (pAs), produced by CuAOs in astrocytes. First, an enhanced gap junction communication can be achieved by pAs [356–358], possibly promoting activity-dependent synchronization [339, 359]. Second, major inhibitory neurotransmitter gamma-aminobutyric acid (GABA) formed from pAs is released by astrocyte-specific GABA transporter [360]. Acting on its extrasynaptic receptor, GABA elevates tonic inhibition and enhances the fast (gamma band) neural oscillations [360]. These ways, the steady-state pA level in astrocytes determined by copper-dependent forming and consuming can be associated with neural circuit activity [244, 255, 362]

Several lines of evidence demonstrate memory deficits concurrent with copper deposition in the choroid plexus, astrocyte swelling (Alzheimer type II cells), astrogliosis and neuronal degeneration in the cerebral cortex, and augmented levels of copper and zinc in the hippocampus of chronically copper-intoxicated rats [337]. Furthermore, these and the other findings concerning the role for astrocytes in brain activity, dis-homeostasis and asscociated diseases [110, 338–341] and brain copper and pA homeostasis in particular [179, 180, 342, 343] may provide support for new astrocyte-centric directions for therapeutic intervention. It can also be depicted by the “gliocentric” alternative of the “neurocentric” STP workflow suggested by Chang [336] possibly associated with major astroglial processes and players of Glu and ammonia homeostasis underlying excitation-inhibition balance in brain [110]. Prevalent traumatic and ischaemic brain injuries are explored to validate the potential of the “gliocentric” concept of early therapeutic intervention. Now, we may add copper-dependent astroglial pA production to the list (Fig. 4**.**) [6, 22, 80, 179, 180, 233, 234, 236–243, 245, 254, 255, 336, 339, 340, 342–362]. GABA can be synthesized from the pA putrescine by copper-containing CuAO in astrocytes. CuAOs perform the oxidation of primary amines such as spermine, spermidine and putrescine to aldehydes and ammonia, producing H_2_O_2_ as a by-product. Putrescine-derived GABA is released by the inside-out (reverse) action of glial GABA transporter subtypes. The increased GABA release and the generated tonic inhibition thereby modulate the power of gamma range oscillation in the CA1 region in vivo. The concentration of cytosolic and extracellular putrescine has been determined to be 22 *nmol/g* and 12 *nmol/g*, respectively [339]. In contrast, copper may decrease tonic inhibition via acting on delta subunit-containing extrasynaptic GABA_A_ receptors [235, 237, 246], thus adding a new layer to disinhibitory mechanisms in copper-rich brain areas.

## Conclusions

Despite multifaceted roles for copper observed in various brain diseases and tumours, the copper signalling theme is still in its initial stages. However, our increasing understanding of dynamic copper pools supports the idea of neuronal activity-dependent Cu(I) transmission affecting astroglia network signaling and astroglia-neuron metabolic cooperation. Rather than simply reflecting copper excess, copper-rich aggregates - likely in astrocytes and not in neurons - are a sign of a disturbed network. Brain diseases linked to oxidative stress [363] change the GSH/GSSG ratio and thereby automatically affect the copper homeostasis, as GSH is the immediate partner, along with various chaperones, that takes Cu(I) from the transporter. Therefore, Cu(I) distribution is disturbed and might in turn enhance oxidative stress at copper-containing deposits or limit Cu, Zn-SOD1 activity in regions with decreased copper level. Closer understanding of copper signalling and its vulnerability opens up new perspectives improving chelate therapy approaches against brain diseases and tumour.

## Abbreviations

AAS: Atomic absorption spectroscopy
AD: Alzheimer’s disease
ALS: Amyotrophic lateral sclerosis
Atox1, CCS, HAH1, COX17: Copper chaperones
ATP7A, ATP7B, Cu-ATPases: Copper-transporting P-type ATPases
Aβ: Amyloid beta
CNS: Central nervous system
CopC: Bacterial peripasmic copper binding proteins
COX, mitochondrial complex IV: Cytochrome c oxidase (EC 1.9.3.1)
CP: *Choroid plexus*
Ctr1, Ctr2: Copper(I) transporters
CTZ: cuprizone (bis-cyclohexanoneoxalyl-dihydrazone)
Cu, Zn-SOD1: Copper, zinc superoxide dismutase
CuAO: Copper aminooxidase
CWD: Chronic wasting disease
DMT1: Divalent metal transporter
D-pen: *D*-penicillamine
EM: Electron microscopy
FAAS: Flameless atomic absorption spectroscopy
GABA: Gamma-aminobutyric acid
GHK: SPARC fragment Gly-His-Lys
GSH: Gluthation (gamma-L-glutamyl-L-cysteinylglycine)
GSSG: Gluthation disulfide
HC: Huntington’s chorea
Htt: Huntingtin protein
ICC: Indian childhood cirrhosis
ICP-AES: Inductively coupled plasma - atomic emission spectrometry
IPs: Inositol phosphates
Kir4.1: Inwardly rectifying K^+^ ion channel 4.1;
LA-ICP-MS: Laser ablation - inductively coupled plasma - mass spectrometry
LDL: Low-density lipoproteins
MD: Menkes disease
MNK1: Menkes protein (soluble cytosolic ATP7A domain)
MT: Metallothionein
μ-PIXE: Micro - particle induced X ray emission
MS: Multiple sclerosis
COMMD1: MURR1 domain protein 1
NMDA: N-methyl-*D*-aspartate
PD: Parkinson’s disease
pAs: Polyamines
PrP^C^: Prion protein, α helical (Adgrg6 receptor agonist)
PrP^Sc^: Prion protein, β sheet enriched (“scrapie”)
SPARC: Secreted protein, acidic and rich in cysteine
STP: Source-target-physiology
SVZ: Sub-ventricular zone
TSPP: Tetrakis-(4-sulfophenyl)-porphine
TM: Tetrathiomolybdate
Trientine: TETA, Trien (Triethylene tetramine)
WD: Wilson’s disease
XFM: X-ray fluorescence microscopy
