# Correction to: Copper signalling: causes and consequences

**DOI:** 10.1186/s12964-018-0292-4

**Published:** 2018-11-12

**Authors:** Julianna Kardos, László Héja, Ágnes Simon, István Jablonkai, Richard Kovács, Katalin Jemnitz

**Affiliations:** 1 Functional Pharmacology Research Group, Institute of Organic Chemistry, Research Centre for Natural Sciences, Hungarian Academy of Sciences, Magyar Tudósok körútja 2, Budapest, 1117 Hungary; 2 Institute of Neurophysiology, Charité-Universitätsmedizin, Berlin, Germany

Following publication of the original article [1], the authors reported an error in Table 3. The correct version of Table 3 is shown below:

**Table 3:** Copper chelating compounds with anticancer activities

Compound type (name)	Structure	Chemical name	Ligand type	Application
TM		Ammonium tetra-thiomolybdate Bis-Choline tetrathio molybdate	Bi-dentate	Breast, prostate, kidney cancer cells [208]
Trientine (TETA, Trien)		N,N′-Bis(2-aminoethyl) ethane-1,2-diamine	Tetra-dentate	Colorectal cancer cells [366]
Hydroxyquinoline (Clioquinol)		5-Chloro-7-iodo-8-hydroxy quinoline	Bi-dentate	AD and human breast cancer cells [309,316]
D-pen		3-Mercapto-D-valine	Bi-dentate	Human leukemia and breast cancer cells [319]
Captopril		D-3-Mercapto-2-methyl-propionyl-L-proline	–	Mammary ductal carcinoma cell line [367,368]
Dithiocarbamates				
Disulfiram (DSF, Antabuse)		1-(Diethylthio-carbamoyl-disulfanyl)-N,N-diethyl-methane-thioamide	–	Human breast, lung cancer cells [315,369]
Pyrrrolidine dithiocarbamate (PDTC)		Pyrrolidine-1-carbodithioic acid	Bi-dentate	Human breast cancer cells [344]
Thiosemicarbazone				
Hydroxyquinoline-carboxaldehyde-Thiosemi-carbazone		8-Hydroxy-quinoline-2-carbox-aldehyde–thio-semicarbazone	R = H tetra-dentate	Prostate cancer cells [370]
Retinal thiosemicarbazone		9-*cis*-Retinal thiosemi-carbazone	Bi-dentate	Human leukemic cell U937 [317]
1,2-Bis(thiosemi-carbazones)		H_2_gts: glyoxal-bis(thiosemi-carbazone) atsm: diacetyl-bis(4-methylthio-semi-carbazone) ptsm: pyruvaldehyde-bis(4-methylthio-semicarbazone)	Tetra-dentate	atsm: human colon cancer tumor cells ptsm: Ehrlich ascites and EMT6 tumour cells [371]
Elesclomol		N’^1^,N’^3^-Dimethyl-N’^1^,N’^3^-bis(phenyl-carbonothioyl)propanedihydrazide	Tetra-dentate	Metastatic melanoma cells[320,331]
Schiff-bases				
Salicylaldehyde-benzoylhydrazone (SBH)		N′-[(2-Hydroxyphenyl) methylidene] benzohydrazide	Bi-dentate [372]	Human adeno-carcinoma cell line [373]
Salicylaldehyde-pyrazole-hydrazone (SPH)		(*E*)- N′-(2-Hydroxy-benzylidene)-1-benzyl-3-phenyl-1H-pyrazole-5-carbohydrazide	–	Lung carcinoma cells [321]
Pyridine-carboxaldehyde-phenylpyrimidyl-hydrazone (Pyimpy)		1-Phenyl-1-(pyridin-2-yl)-2-(pyridin-2-ylmethylene)hydrazine	Tri-dentate	Rat breast tumor cells [322]
Hydroxy naphthaldehyde imine (HL)		1-(((2-((2-Hydroxy-propyl)amino) ethyl)imino) methyl) naphthalene-2-ol)	Tri-dentate	Human cervical and liver hepatocellular carcinoma cells [318]

The publishers apologise for this error. The original article [1] has been corrected.
